# Supplementary material for: Impact of the COVID-19 Pandemic on the Evolution of Prevalence and Patterns of Cannabis Use among First-Year University Students in Spain—UniHcos Project
Source: Int J Environ Res Public Health. 2022 Sep 14;19(18):11577. doi: 10.3390/ijerph191811577 (PMC9517240; doi:10.3390/ijerph191811577)

Table S1. Tobacco and alcohol use characteristics during the periods of study

	Period of Study		
	**Before COVID-19**	**Lockdown**	**New Normal**
	**N(%)**	**N(%)**	**N(%)**
**Tobacco**			
Non-smoker			
Total	6780 (72.6)	457 (74.0)	420 (73.8)
Men	1871 (74.1)	102 (72.9)	107 (74.3)
Women	4909 (72.1)	355 (74.3)	313 (73.7)
Ex-smoker			
Total	682 (7.3)	63 (10.2)	49 (8.6)
Men	178 (7.1)	11 (7.9)	18 (12.5)
Women	504 (7.4)	52 (10.9)	31 (7.3)
Current smoker			
Total	1873 (20.1)	98 (15.9)	100 (17.6)
Men	477 (18.9)	27 (19.3)	19 (13.2)
Women	1396 (20.5)	71 (14.9)	81 (19.1)
**Alcohol**			
Past-30 days			
Total	7334 (78.6)	394 (63.8)	442 (77.7)
Men	2040 (80.8)	89 (63.6)	106 (73.6)
Women	5294 (77.8)	305 (63.8)	336 (79.1)

Figure S1: Percentage of university students starting cannabis use by age and period of study.

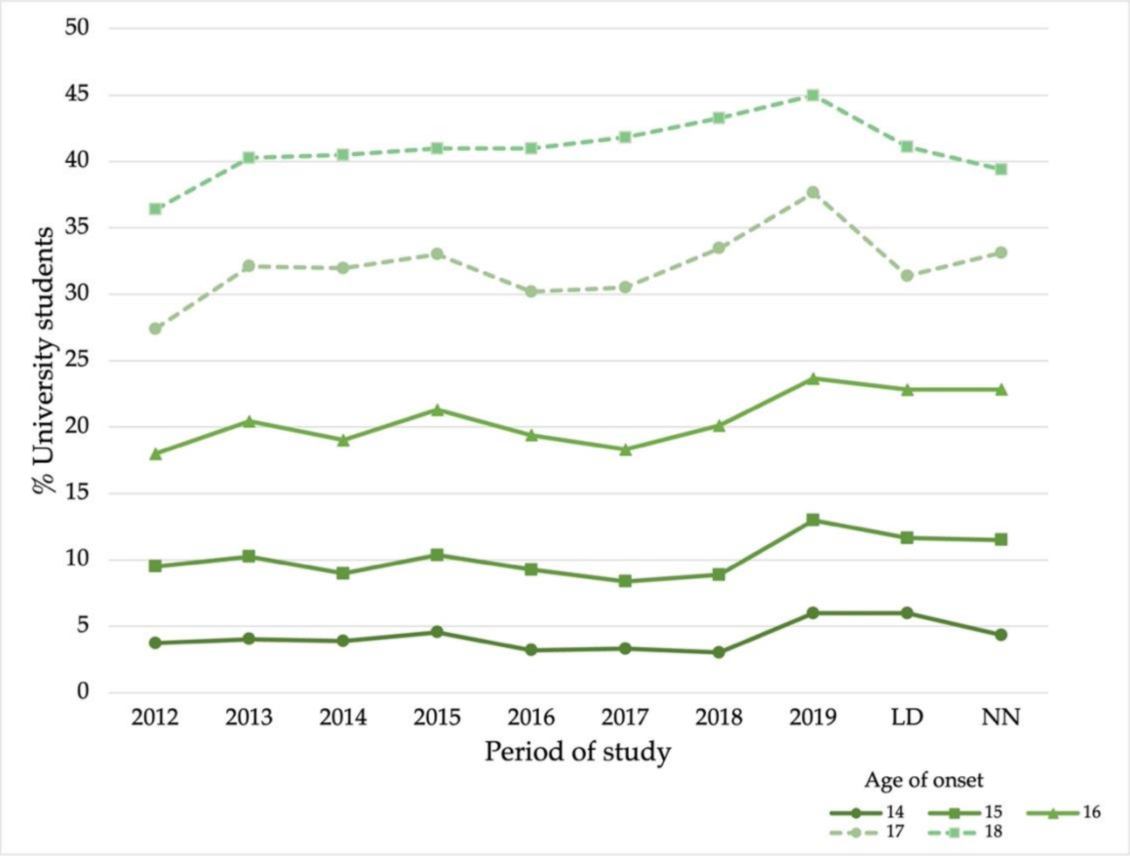

Supplement: Supplementary file 1 [file ijerph-19-11577-s001.zip › ijerph-1861420-supplementary.pdf]
